# Purification and study of anti-cancer effects of *Serratia marcescens* serralysin

**Published:** 2019-08

**Authors:** Almas Araghi, Saba Hashemi, Abbas Akhavan Sepahi, Mohammad Ali Faramarzi, Mohsen Amin

**Affiliations:** 1 Department of Microbiology, Faculty of Biological Sciences, North Tehran Islamic Azad University, Tehran, Iran; 2 Department of Pharmaceutical Biotechnology, School of Pharmacy, Tehran University of Medical Sciences, Tehran, Iran; 3 Department of Drug and Food Control, School of Pharmacy, Tehran University of Medical Sciences, Tehran, Iran; 4 The Institute of Pharmaceutical Sciences (TIPS), Tehran University of Medical Sciences, Tehran, Iran

**Keywords:** *Serratia marcescens*, Serralysin, Purification, Ion exchange chromatography, Anti-cancer

## Abstract

**Background and Objectives:**

Serralysin is an extracellular metalloprotease from *Serratia marcescens* which has been the subject of extensive biological investigations. The goal of this study was to extract and purify serralysin from *S. marcescens* and to investigate its cytotoxic activity on the colorectal cancer cell line.

**Materials and Methods:**

The presence of the serralysin gene was confirmed using PCR. The supernatant of bacterial culture was collected and precipitated using ammonium sulfate. The precipitated protein was dialyzed and subjected to ion exchange chromatography for further purification. Casein assay and skim milk assay was used to confirm the enzymatic activity. SDS-PAGE was used to visualize the presence of serralysin. Metalloprotease inhibition activity was performed using 50 mM EDTA. Cytotoxic activity of serralysin was assessed on MTT assay.

**Results:**

The PCR product corresponding to serralysin was estimated to be approximately 1500 bp. A transparent zone around the bacterial colonies on skim milk agar and casein digestion confirmed the proteolytic activity of serralysin. A 52 kDa band in SDS-PAGE corresponding to serralysin was observed before and after purification processes. MTT assay showed IC_50_ values 24.78 μg/ml and 19.16 μg/ml after 24 h and 48 h exposure of Caco-2 cells to serralysin, respectively.

**Conclusion:**

Our results showed that native serralysin has anticancer potential and may be a candidate for further pharmaceutical research and development. Further *in vivo* and *in vitro* mechanistic studies are suggested to confirm the biological activities.

## INTRODUCTION

Colorectal cancer is one of the notorious cancers in terms of incidence and mortality. Over 1.8 million new colorectal cancer cases and 881,000 deaths are estimated to occur in 2018 (1). There is a growing body of evidence on the use of secondary microbial metabolites such as pigments, bacteriocins, and toxins for the treatment of cancers (2). Production of various proteases has been reported from different strains of *Serratia* with caseinolytic activity (3).

*Serratia marcescens* is a Gram-negative, facultative anaerobeic, rod-shaped bacterium and an opportunistic nosocomial pathogen. The bacterium is capable of secreting many hydrolytic enzymes such as proteases, lipases, chitinases, hemolysins, and nucleases which can be used in certain industrial and medical processes (4). *S. marcescens* secrets a metal-loprotease called serralysin with proteolytic activity. Serralysin is also known as serrapeptidase, serratiopeptidase, and serratia peptidase. It has been purified from *S. marcescens* E-15 inside a silkworm gut for the first time (5). Serralysin-derived drugs have been commercially available under brand names Serrapeptase and Danzen for anti-inflammatory purposes (6). Serralysin has found various applications in food, leather, textile, and pharmaceutical industries. In this context, identification of serralysin-producing microorganisms and the method of the enzyme purification is of great importance. Although many studies have revealed the anti-inflammatory effects of serralysin, the exact anti-inflammatory mechanism is still unknown. Research findings have shown that serralysin dissolves the dead and damaged tissues which are by-products of the healing process without harming normal tissues (7). Chronic inflammation causes a variety of diseases, such as cardiovascular disease, pulmonary disease, autoimmune diseases, and cancer (8). In recent years, cancer patients have shown resistance to chemotherapy which requires devising new therapeutic approaches. The ideal cancer treatment is to target cancer cells exclusively with the least toxic effects on normal surrounding tissues. The use of enzymes and toxins of microorganisms has been recognized as potential therapeutic means in the past decade (9). Many viruses (vaccinia, newcastle, retroviruses, and adenoviruses) have a strong tendency to multiply in cancerous cells and to eliminate them. Also, bacteria have been shown to produce toxins and eliminate selective cancer cells (10–12).

Chronic inflammation and the presence of bacterial biofilms in the colon are important risk factors for the progression of colon cancer (13). Serralysin is a natural protease that attaches to the alpha-1 macroglobulin (α_1_M) in the blood and evades from the immune system via molecular camouflaging while its enzymatic activity is maintained and is slowly transmitted to the inflammation site. It hydrolyzes bradykinin, histamine, and serotonin that are responsible for inflammation. It can inhibit the increasing amount of fibrin, thus cancer cells cannot bind to proteins and subsequently cannot enter the bloodstream (14). Bacterial biofilms are associated with a collection of bacteria that cause damage to the tissue and alter the tissue homeostasis and increase cell proliferation (15). Generally, serralysin increases the antibacterial effect against biofilm-forming bacteria that can cause colon cancer through the moderating of adhesion in certain bacteria (16, 17). The idea that proteinases eliminate solid tumors has led to the discovery of a new group of drugs that can eliminate solid tumors in cancerous tissue and can destroy cells with uncontrolled reproduction. For some unknown reason, proteases cannot kill all types of cancer cells and the mechanism of action is correlated with the type of tissue around the tumor, especially when the tumors are advanced and deep (17). Since inflammation is one of the factors that play role in colon cancer, and is the cause of many deaths in the world, in this study we investigated the anticancer effects of serralysin using Caco-2 cells as a model cell.

## MATERIALS AND METHODS

RPMI-1640 medium was purchased from Biosera, England, fetal bovine serum (FBS), Dulbecco’s modified Eagle’s medium (DMEM), penicillin – streptomycin, trypsin, trypsin – EDTA were purchased from PAA Laboratories, Austria. 3-(4,5-Dimethylthiazol-2-Yl)-2,5-Diphenyltetrazolium Bromide (MTT), Bovine serum albumin (BSA), trypan blue dye, ammonium sulfate, Dimethyl sulfoxide (DMSO) were purchased from Sigma Aldrich, USA. β- Mercaptoethanol, sodium dodecyl sulfate, tris (hydroxymethyl) aminomethane, glycine, hydrochloric acid, sodium carbonate, Skim milk powder, Muller Hinton broth, peptone, agar, agarose, and acrylamide were purchased from Merck, Germany.

### Bacterial strain and growth conditions.

The bacterial strain of *S. marcescens* ATCC 14756 was provided by Pasteur Institute of Iran, Tehran. Bacteria were inoculated into the Muller Hinton broth in an orbital shaker incubator at 37°C and 140 rpm. Bacterial growth was measured by optical density (OD) of the cell suspension at 600 nm during a week. Bacterial growth was measured during one week twice a day.

### Molecular detection of serralysin gene.

PCR was used to detect the serralysin gene. We also searched in the NCBI BLAST server (http://www.ncbi.nlm.nih.gov/BLAST) to find sequence homology of the serralysin gene. The forward primer: 5′-ATG CAA TCT ACT AAA AAG G-3′, and reverse primer: 5′-TTA CAC GAT AAA GTC AGT G-3′ were designed and checked in the MUSCLE or MAFFT-multiple sequence alignment program and were investigated in the NCBI server. The genome of *S. marcescens* strain ATCC 14756 was prepared by boiling-lysis and PCR program mentioned as follow: pre-denaturation at 95ºC for 5 min, denaturing at 94ºC for 1 min, annealing at 60ºC for 45 s, extension at 72ºC for 1.5 min (35 cycles) and a final polymerization step at 72°C for 10 min. PCR product was loaded in a 1% agarose gel.

### Serralysin production.

*S. marcescens* was grown at 100 ml LB media until bacterial growth reached OD = 1 at 600 nm and then was inoculated into 1000 ml LB medium at 37°C on a shaker at 140 rpm.

### Serralysin partial purification and dialysis.

Protease partial purification was performed by centrifuging of 1000 ml of bacterial culture at 5,500 rpm for 30 min at 4°C and the supernatant of this step was precipitated with (20%, 35%, 50%, 65%, 80%, and 85%) ammonium sulfate saturation. Due to the supernatant volume, the amount of the ammonium sulfate was calculated and added slowly at 4°C in 6 h to the supernatant and then it was centrifuged at 11,000 g for 20 min. The pellet, collected by dissolving in 50 mM Tris buffer (pH 8) and then dialysis performed against the same buffer for 48 h at 4°C, the buffer was changed three times.

### Ion exchange chromatography.

Ion exchange chromatography is a method of separating and purifying proteins and other charged biomolecules. To load the sample onto the Q-Sepharose column, subsequent steps were carried out at 4°C, and the column was equilibrated with 50 mM Tris buffer pH 8. After sample loading, NaCl was used with different gradient concentrations of 0 to 1400 mM. The absorbance of 1 ml of each elution was measured at 280 nm. Protease activity and SDS-PAGE were carried out subsequently.

### Polyacrylamide Gel Electrophoresis (SDS-PAGE).

Polyacrylamide gel electrophoresis was performed using Laemmli method (19) with lower separating gel (pH 8.8) and upper stacking gel (pH 6.8). Samples (∼20 μl) were mixed in loading buffer and after boiling for 15 min, were electrophoresed on a 12% polyacrylamide gels concentration. The serralysin molecular weight was compared with the Protein marker. The protein bands were visualized by the staining of the Coomassie Blue G-250. The protein concentration was measured with the Bradford method with bovine serum albumin as a reference.

### Protease assay.

In order to measure the activity of proteases, Sigma’s non-specific protease activity assay can be accomplished. In this assay, casein acts as a substrate and after digesting casein by protease, tyrosine with other amino acids and peptide fragments is liberated. Folin’s reagent primarily reacts with free tyrosine and the blue color resulting from this reaction is quantifiable by spectrophotometer (22). Skim milk assay and casein assay were used to determine protease activity before and after purification. Skim milk agar plate was used and 10 μl of samples were applied at different spots on the agar. Clear zones over time demonstrated protease activity. Casein assay was carried out with a reaction solution including 500 μl of sample and 1000 ml of 50 mM Tris-HCl buffer pH 8.0 and incubated for 15 min at 40°C. To discontinue the reaction, 500 μl trichloroacetic acid (TCA) was added to the solution and centrifuged at 8000 rpm for 10 min, and folin and ciocalteus phenol plus Na_2_CO_3_ was added to the solution and after 10 minutes OD was measured at 660 nm.

### Protease inhibition.

To confirm the metalloprotease activity, 100 μl of serralysin and 100 μl of 50 mM EDTA were mixed. The mix of 100 μl of serralysin and 100 μl of ddH_2_O was used as a negative control. The mixes were used in the skim milk agar assay.

### Cytotoxicity assay of serralysin on Caco-2 cancer cell line.

Caco-2 cells, the human colonic epithelial cancer cell line was provided by the cell bank of the Department of Toxicology, Faculty of Pharmacy, Tehran University of Medical Sciences. To determine the viability of the cancer cell line, the MTT colorimetric assay was carried out. Caco-2 cells were grown in 50% RPMI 1640, 34% DMEM-F12, 15% FBS, and 1% penicillin-streptomycin in a 25-cm^2^ plastic flask in humidified atmosphere of 5% CO_2_ at 37°C for 48 and 72 hours. Caco-2 cells were detached from flasks by trypsin and trypsin was neutralized with PBS. Cell suspension colored by 0.4% trypan blue and alive cells were counted by hemocytometer chamber. Caco-2 cells were cultured at a density of 1 × 10^3^ per 96-well plates and after attachment, cells were incubated with different concentrations of the enzyme (ranging from 1.25 to 25 μg/ml) for 24 h and 48 h. MTT 3-(4,5-dimethylthiazol-2-yl)-2,5-diphenyltetrazolium bromide was added for 3-4 h until formazan reacted with dehydrogenase of viable cells and then was removed by 100 μL DMSO, the absorbance was measured at 570 nm and the data were analyzed by GraphPad Prism 7.

## RESULTS

### Molecular detection of serralysin gene in *S. marcescens*.

Serralysin was first isolated from *Serratia* E-15 by Kodoma et al. (5). To amplify the serralysin gene, DNA extraction was performed and PCR was carried out using the designed primers and approximately a 1464 bp gene was observed in %1 agarose gel (data not shown).

### Growth curve.

**Fig. 1:**
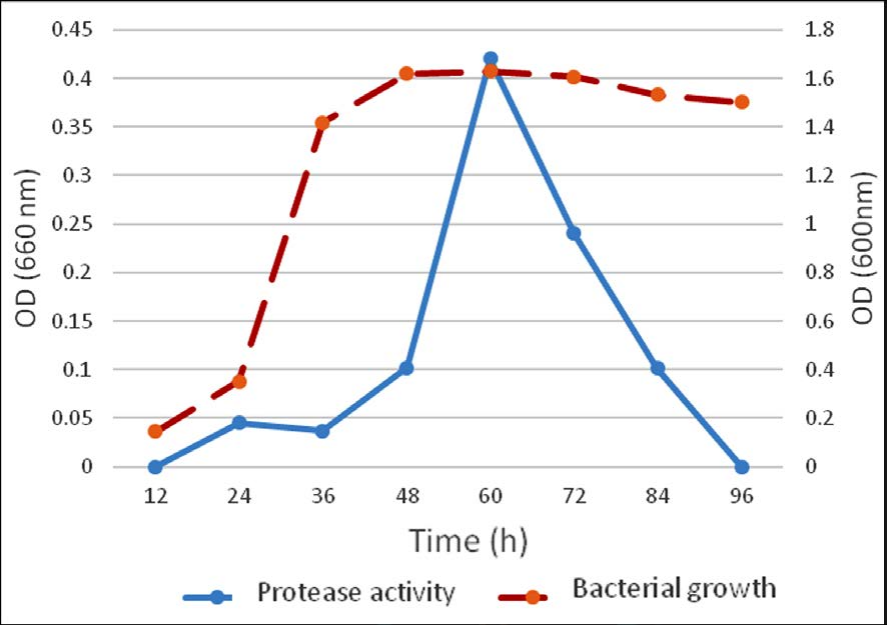
Bacterial growth and protease activity. Bacterial growth curve of *S. marcescens* with lag, log, and stationary phases was measured at 600 nm. Maximum protease activity was observed 60 h after bacterial growth at 660 nm.

The number of microorganisms in a culture medium expands exponentially as long as the nutrient is consumed and at each phase bacteria produce metabolites, therefore population size measurement is important against time. One colony of *S. marcescens* was inoculated into 5 ml Muller Hinton broth medium and incubated at 37°C with 140 rpm until bacterial growth was measured OD = 1 at 600 nm. 0.5 ml of bacterial suspension was inoculated into 50 ml LB medium and incubated at 37°C with 140 rpm for a week. Bacterial growth and protease activity were measured during one week twice a day (Fig. 1). In order to obtain an optimal amount of enzyme production, optimal nutrient sources and environmental conditions should be considered.

### Protease assays.

Protease activity was investigated by two methods: 1. In skim milk agar plates we observed clear zones of proteolysis around the *S. marcescens* colonies and 2. Casein assay determined protease activity and the results of this assay curve was converted to μM by tyrosine standard curve which is shown in (Fig. 1). Maximum protease production was observed at stationary phase of bacterial growth (Fig. 1). The supernatant SDS-PAGE of *S. marcescens* was carried out 60 h after bacterial growth (data not shown).

### Purification.

To achieve high purity protein, ion-exchange chromatography was used. After partial purification stages including precipitating by 80% ammonium sulfate and dialysis, SDS-PAGE analysis was confirmed and approximately a 52 kDa band was observed and casein assay was performed.

**Fig. 2:**
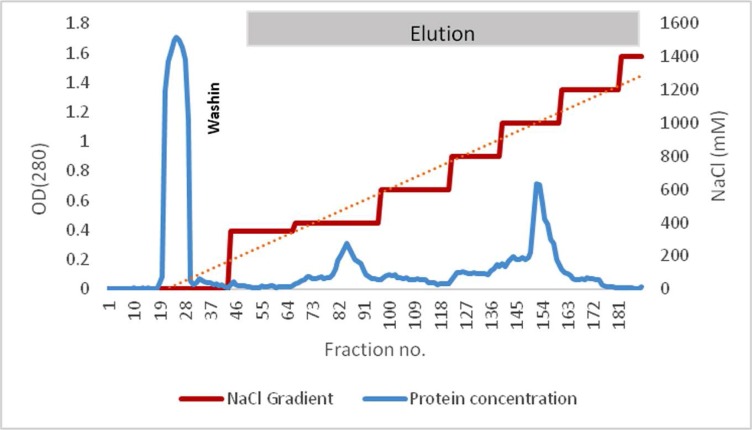
Protein absorption at 280 nm with different NaCl concentrations. Maximum OD was observed at 85 and 152 fractions which were eluted by 400 mM and 1000 mM NaCl, respectively.

After precipitation, crude protease was loaded onto the Q-Sepharose column which was equilibrated by Tris pH 8 and all fractions were collected in 1 ml volume. The protease was eluted by different concentrations of NaCl solution and protease optical density per fraction was assessed at 280 nm (Fig. 2).

**Fig. 3:**
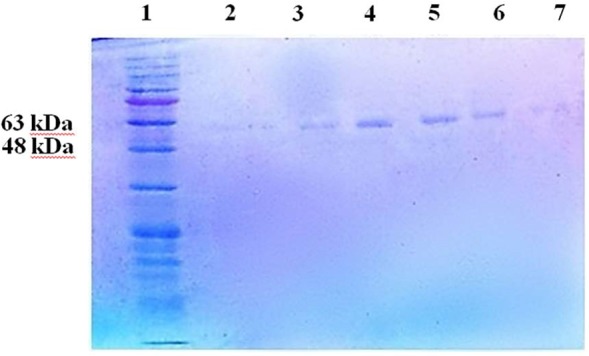
SDS-PAGE after ion exchange chromatography. A 52 kDa bands corresponding to fractions with maximum OD at 280 nm were observed. 1) Protein ladder, 2) fraction no.83, 3) fraction no.84, 4) fraction no.85, 5) fraction no.86, 6) fraction no.87, 7) fraction no. 88

The fractions were analyzed by SDS-PAGE and approximately a 52 kDa protein was observed with casein activity. In this study, there were two peaks in fraction number 85 and fraction number 152 that was observed from fraction number 83 to fraction number 88, single bands of the enzyme with a molecular weight of approximately 52 kDa (Fig. 3).

### Metalloprotease inhibition assay.

Protease activity of fractions number 83 to fraction number 88 was inhibited by 50 mM EDTA in skim milk agar plates with no clear zones caused by casein digestion. This experiment confirms the proteolytic activity of serralysin.

### Cytotoxicity of serralysin on Caco-2 cancer cells.

**Fig. 4:**
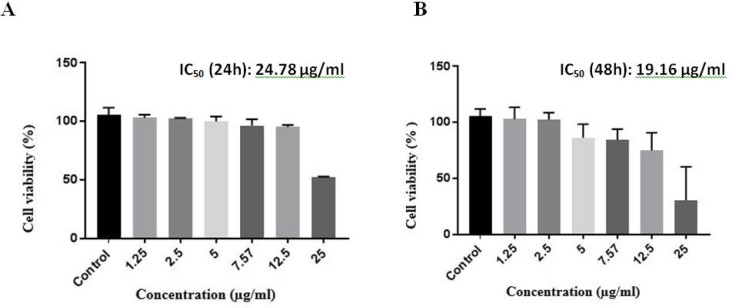
A) Assessment of serralysin cytotoxicity on Caco-2 cells by MTT assay. The graph corresponds to 24 h exposure of serralysin to 10,000 Caco-2 cells per well. IC_50_ was determined 24.8 μg/ml after 24 h treatment. B) Assessment of serralysin cytotoxicity on Caco-2 cells by MTT assay. The graph corresponds to 48 h exposure of serralysin to 10,000 Caco-2 cells per well. IC_50_ was determined 19.16 μg/ml after 48 h treatment.

Cytotoxicity of serralysin was assessed on colon cancer cell lines (Caco-2) using MTT assay. The cytotoxicity profile was outlined after 24 h and 48 h by treating serralysin on Caco-2 Cell lines. Caco-2 cells were cultured at a density of 10^4^ per well. IC_50_ values after 24 h and 48 h treatment was 24.78 μg/ml and 19.16 μg/ml, respectively (Fig. 4). The IC_50_ values were determined using GraphPad Prism 7.

## DISCUSSION

According to the studies, tryptone is the key source of nitrogen for *S. marcescens* to produce serralysin and certain carbohydrates such as glucose, lactose, maltose, sucrose, xylose, glycerol, mannitol and sorbitol have been reported as carbon sources for *S. marcescens* (20). In a study, in order to obtain high production of *S. marcescens* ATCC 25419 serralysin, a medium containing casein, peptone, tryptone and skim milk was used as inducers and protease activity began at the early stages of growth and the highest protease activity was observed in the stationary phase and in the late stationary phase enzymatic activity was constant. In this study, tryptone/peptone was used and maximum constant activity at the stationary phase was observed (21).

In two studies, different methods were used to purify serralysin. In a study to purify this enzyme, supernatant sedimentation of *S. marcescens* ATCC 25419 with ammonium sulfate was used in the range of 50–70%, and dialysis was performed against Tris-HCl buffer (pH 8). The sample was loaded onto the Q-Sepharose column and was washed with 400 NaCl mM then the samples with maximum protease activity were precipitated with 80% ammonium sulfate and was passed through the S-200 column. Using this method, a metalloprotease with a molecular weight of 53.5 kDa and a protease was purified with a molecular weight of 56.6kD (18).

In 1984, Matsumoto et al. purified four proteases from *S. marcescens* kums 3958 using ammonium sulfate precipitation, DEAE ion exchange chromatography and Sephadex filtration gel (11). The results of their agarose gel showed three proteases with molecular weights of 56.60 and 73 kDa.

In the present study, the standard strain of *S. marcescens* was used to purify serralysin with 80% concentration of ammonium sulfate, dialysis, and Q-Sepharose ion exchange chromatography column. The protease enzyme was purified with approximately a molecular weight of 52 kDa, and the purified enzyme was used for cytotoxicity assessment on Caco-2 cells.

William Coley was one of the pioneers to report on the anti-cancer effects of *Streptococcus pyogenes* in patients with neck cancer. In the 1800s, he used two species of *Streptococcus pyogenes* and *S. marcescens* to prepare a vaccine against sarcoma, carcinoma, melanoma, and lymphoma (12). In 2016, according to studies by Gao et al. the effect of dose-dependent toxin fungus on Caco-2 cancer cell line was investigated and showed that aflatoxin M1 and ochratoxin are more toxic than zearalenone and α- zearalenol (Table 1) (23). The reason for this difference in the results was the difference in the type of cell lines. In a study by Sam Mather in 2005, the cytotoxic effect of several antimicrobial peptides was considered. Maher et al. reviewed the effect of cytotoxic antimicrobial peptides including magainin, nisinA gallidermin, melittin and antibiotic vancomycin on cell lines of HT29 and Caco-2. In this study, the IC_50_ of each compound was mentioned for the two cell lines and was measured by MTT assay. Accordingly, IC_50_ of these compounds on Caco-2 cell line was reported as gallidermin (21.5 μm), vancomycin (more than 6000 μM), Magainin I (65 μM), Magainin II (85 μm), nisin (9 / 89 μmolar) and melittin (1.2 μm). Thus, the melittin cytotoxicity rate in this study is higher than other compounds studied on Caco-2 cell line (25). In 2016, Noura El-Ahmady El-Naggar et al. investigated the antitumor effects of the asparaginase enzyme produced by *Streptomyces fradiae* NEAE-82 on HepG2, Hep2, and Caco-2 cells. In these studies, it was found that purified asparaginase have cytotoxic effects on cancerous cell lines. The anticancer effect on Caco-2 cell line was greater than the other two cell lines, and it was found that 4 IU of purified asparaginase (equal to 22 μg/ml) could stop the growth of the Caco-2 cell line. In that study, it was found that the required temperature, incubation time, and optimal pH to reach the maximum asparaginase activity were 40°C, 30 minutes and 8.5, respectively (26). According to the results of this study, IC_50_ of serralysin on Caco-2 cancer cell line was 24.78 μg / ml at 24 h and 19.6 μg/ml at 48 h.

**Table 1.** IC_50_ comparing of metabolites with anti-cancer activity on Caco-2 cell line.

**Anti-cancer agent**	**IC_50_**	**Cell line**	**Incubation time (h)**	**Reference**
OTA (ochratoxin A)	2.07 μM	Caco-2	72	(23)
AFM1 (Aflatoxin M1)	4.10 μM	Caco-2	72	(23)
ZEA (zearalenone)	11.62 μM	Caco-2	72	(23)
α -ZOL (α- zearalenol)	22.90 μM	Caco-2	72	(23)
Nisin A	115 μM	Caco-2	24	(25)
Gallidermin	210.5μM	Caco-2	24	(25)
Asparaginase	22 μg/mL	Caco-2	48	(26)
